# Taguchi Optimization of Wetting, Thermal and Mechanical Properties of Sn-1.0wt.%Ag-0.5wt.%Cu Alloys Modified with Bi and Sb

**DOI:** 10.3390/ma17112661

**Published:** 2024-06-01

**Authors:** Sung-joon Hong, Ashutosh Sharma, Jae Pil Jung

**Affiliations:** 1 Department of Materials Science and Engineering, University of Seoul, 163, Seoulsiripdae-ro, Dongdaemun-gu, Seoul 02504, Republic of Korea; joon337@naver.com; 2 Department of Materials Science and Engineering, Ajou University, Suwon 16499, Republic of Korea; 3 Amity Institute of Applied Sciences, Amity University Jharkhand, Ranchi 834002, India

**Keywords:** lead-free solder, SAC alloy, wetting, microstructure, mechanical properties

## Abstract

This study was conducted on SAC105 (Sn-1wt.%Ag-0.5wt.%Cu) lead-free solder modified with Bi and Sb. The wetting, melting point, and mechanical properties were analysed with the addition of 1~5 wt.%Bi and 1~5 wt.%Sb for SAC105 base alloy. The wetting characteristics were assessed by wetting time (zero cross time, ZCT) obtained from wetting balance tests. The mechanical properties were analysed by tensile tests. Considering two factors (Bi, Sb), a three-level (0, 1, 2 wt.%) design of experiment (DOE) method array was applied for Taguchi optimization. The results indicated that the solder wetting increased as Bi content increased, while it decreased with Sb. The ZCT decreased with increasing Bi content up to 4 wt.%, while it increased proportionally to Sb content. The melting point, measured using a differential scanning calorimeter (DSC), showed that the melting point tended to decrease according to Bi increase, while it increases depending on the Sb content. Increase in Bi and Sb levels resulted in enhanced tensile strength in the mechanical properties tests, with Bi having a more noticeable impact. The Taguchi optimized conditions for the Bi and Sb studies were found to be 2 wt.%Bi and 2 wt.%Sb. This led to an optimal set of 0.9 s of wetting time, a 222.55 °C melting point, a 55 MPa tensile strength, and a 50% elongation.

## 1. Introduction

The rapid advancement of the contemporary electronics market has heightened the need of high speed, potable, durable, and highly reliable solder joints [1]. In recent years, concerns about the elevated toxicity of lead (Pb) have prompted researchers to explore various Pb-free solder alternatives to substitute for the conventional Pb-bearing solders [2,3]. The European Union banned the use of Pb in solder alloys as part of the 2006 Restriction of Hazardous Substances (RoHS) and Waste Electrical and Electronic Equipment (WEEE) directives, and many other countries around the world have since followed this actively. After such numerous worldwide regulations over the use of Pb in electronic devices, the development of Pb-free soldering alloys excluding the Pb composition that satisfy the microjoining operations and solder joints has emerged.

These Pb-free solder options encompass Sn-Cu, Sn-Ag, Sn-Zn, Sn-Ag-Cu, Sn-Bi, Sn-In, and other alternatives [4,5,6,7,8]. The goal in developing Pb-free solders is to ensure comparable or superior performance compared to Pb-bearing solders. Factors considered in the formulation of Pb-free solders include eco-friendliness, material availability, and economy. Most importantly, a replacement should be a better substitute to conventional Sn-Pb solder in terms of wetting, melting point, density, electrical properties, bonding strength, and oxidation characteristics. Among the various Pb-free options available, eutectic Sn-3.0Ag-0.5Cu (SAC305) solder alloys are potent Pb-free alloys over binary alloys due to their exceptional wetting, mechanical, and tensile properties, as well as their excellent compatibility with existing reflow and flux chemistry. Currently, SAC305 is the standard Pb-free solder alloy for microelectronic packaging components [2,3].

Nevertheless, all these SAC alloys exhibit elevated Ag content in their components which is not desired due to the continuous rising of Ag price globally. To improve the cost-effectiveness, low Ag SAC alloys have garnered significant interest due to their increased drop impact reliability [9]. However, SAC alloys with low Ag content typically demonstrate relatively depressed wetting, lower resistance to thermal loading due to reduced formation of strengthening intermetallic compound (IMC) phase, specifically Ag_3_Sn [10,11].

Considerable efforts have been invested in enhancing the properties of Pb-free SAC solders. Introducing alloying additions is a proven strategy for improving the soldering characteristics of SAC alloys. The various alloying elements used in SAC alloys are Cu, Ag, Bi, Sb, In, Al, etc. [4,5,6,7,8,12]. Some of the researchers have also used ceramics (SiC, CeO_2_, ZrO_2_, La_2_O_3_, TiO_2_, ZrSiO_4_, etc.) [1,9,13,14,15,16] to improve the properties of SAC alloys. Novel nanomaterials such as graphene, carbon nanofibers, boron nitride nanotubes, etc. have also been explored to enhance the wetting and mechanical performance of SAC alloys [17,18,19]. However, the ceramic additives have an issue of segregation and de-wetting during reflow operations [20]. Among the metallic additives, recently Sb has been validated as an effective alloying element capable of hindering the evolution of large β-Sn and Ag_3_Sn IMC needles while improving the thermal resistance of SAC solders [21,22]. Bi is another pivotal alloying element used in solder alloys to study their hardness and creep properties [23,24]. It is well documented that the addition of Bi in Sn has a room temperature solubility limit of 1.8 wt% [25]. Therefore, Bi will be segregated when it is present in an amount greater than 1.8 wt% in a Sn matrix and increase the resistivity of the alloy. Previous studies also showed that addition of Bi greater that the solubility limit raises the electrical resistivity of Sn-solder alloys [26]. As reported by Kisiel et al. for alloys with 3 wt.%Bi and Sb, the resistivity is comparable to those of Pb-Sn solders (14.44 µΩ.cm) which is good for functioning of brazed electrical devices [27]. Additionally, limited research exists which studies the combined effects of dual additive elements (Bi, Sb) on the microstructures and properties of low-Ag solder alloys. Thus, this study systematically investigates the addition of Bi and Sb to the low-Ag Sn-1.0Ag-0.5Cu (SAC105) alloy, analysing their influence on melting properties, wetting, microstructure, and mechanical characteristics.

It is also a noteworthy point that despite all these developments in Pb-free solder research, the achievement of the best set of soldering properties requires a homogeneous dispersion of additive phases in the matrix. The enhancement in soldering properties of the material is due to the suppression and distribution of needle shaped Ag_3_Sn IMCs, wetting of the additive to the matrix, hardness, and strength, which contribute towards the service life of an electronic packaging system. Thus, to have a deeper understanding, we have used the Taguchi model and analysis of variance (ANOVA) technique to predict appropriate process parameters for low silver SAC105/(Bi, Sb) system. In this work, we chose two factors Bi and Sb additive elements and their three-level concentration (0, 1, 2 wt.% each). A general full factorial DOE was applied, and extended levels of factors were applied to confirm the tendency of each property.

## 2. Experiment

In this Section, we describe the detailed experimental procedures and characterization studies undertaken to investigate the effects of Sb and Bi additions on the properties of SAC filler metal. This study aims to understand how varying concentrations of Bi and Sn influence microstructure, wetting, melting point, and tensile stress of the SAC105 alloy for soldering applications. The experiments were carefully designed to provide comprehensive insights into the material characteristics under different additive combinations and concentrations as discussed in the following subsections.

### 2.1. Synthesis, Microstructure, and Thermal Analysis

SAC105 alloy was incorporated with Sb and Bi (in wt.% from 0 to 5) to fabricate SAC105/Bi, SAC105/Sb, and SAC105/(Bi + Sb) composite solders. The particle sizes of SAC105, Bi, and Sb powder was type 4 (20–38 µm), 150 µm, and 60 µm, respectively. The mixing of matrix alloy (SAC105) with Bi and Sb powder was conducted in a low energy ball mill at 200 rpm with a 5:1 ball-to-powder weight ratio in an Ar atmosphere. Ball milling was carried out for 4 h in a ball mill (RETSCH PM-400, Haan, Germany) using toluene as the process control agent. RMA flux was added to the powder mixture in a ratio of 9:1 to form a well-dispersed solder paste. The solder pastes were reflowed at 280 °C to a obtain well-dispersed bulk solder alloy in a crucible (Φ15 mm × 5 mm). Table 1 shows the number of samples with varying Bi, Sb and Bi + Sb prepared through the mechanical mixing and melting method.

Microstructure analysis was conducted through scanning electron microscopy (SEM, Hitachi 4800S, Tokyo, Japan). All the images were captured in secondary electron mode. The composition of solders was determined by energy dispersive spectroscopy (EDS) outfitted with the SEM machine. The phase analysis of the samples was conducted using an X-ray diffractometer (XRD, Brukers D8 Advance, Karlsruhe, Germany) with a Cu target (K_α_ = 1.54 Ǻ). Thermal analyses of the prepared solder specimens were carried out using a differential scanning calorimeter (Diamond DSC Perkin Elmer, Bridgeport Avenue, Shelton, CT, USA) at a scanning rate of 10 °C/min from 50–250 °C. All tested samples were contained in hermetically sealed Al pans. The in-built software program was used to compute the peak melting temperatures of the specimens.

### 2.2. Wetting Balance Test

**Figure 1:**
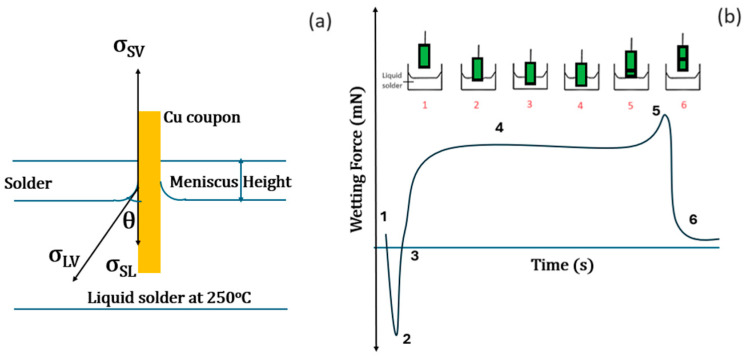
Wetting balance test. (**a**) Scheme showing various forces acting on the coupon–solder interface. (**b**) Wetting balance curve showing various position of meniscus at different instants. The numbers 1 to 6 show different positions of the coupon when it is immersed and withdrawn from the solder bath. Accordingly, the respective change in wetting force is traced with respect to time and the numbers are displayed in wetting force curve corresponding to the positions 1 to 6.

The wetting property of the SAC105 alloys in this work was investigated using a wetting balance tester (RHESCA SAT 5000 series, Tokyo, Japan). A Cu plate (oxygen-free Cu, 99.9% pure, 10 mm × 30 mm × 0.3 mm), served as the substrate for wettability measurements. The Cu coupon was prepared through mechanical grinding and polishing with SiC emery papers (Grit #2000 and #2400) and subsequent ultrasonication to eliminate the inherent oxide and foreign particles. Afterward, the Cu specimen was rinsed with 5% HCl-95% C_2_H_5_OH for 15 s to eliminate any greasy layer. Before the wettability tests, solder flux (Sparkle Flux WF-6063M5, Senju, Japan) was lightly applied to coat the Cu coupon, followed by activation through warming over a solder bath for 30 s. The Cu specimen was then dipped down to 2 mm in solder bath maintained at 250 °C at a speed of 2.5 mm/s for 5 s. The measurements were conducted by observing the wetting force and time to wet the solder Cu specimen during immersion in the solder bath [12]. The scheme depicting the wetting balance test is shown in Figure 1a,b. Figure 1a shows the various interfacial tension forces acting on the liquid solder Cu coupon is immersed into it. The change in the Cu coupon position from position 1 to 6 during immersion process and respective measured wetting forces at these positions are depicted in Figure 1b.

From Figure 1a, the equilibrium wetting angle subtended at the interface is given using the interfacial tensions, *σ_SV_* (solid–air), *σ_SL_* (solid–liquid), and *σ_LV_* (liquid–air), employing the Young–Dupre equation [12]:

*σ_SV_* − *σ_SL_* = *σ_LV_*cos*θ* (1)

The meniscus height exhibits a direct proportionality to the wetting force (*F*_net_) exerted by the liquid solder onto the Cu coupon given by (2).

*F*_net_ = *pσ_LV_*cos*θ* − *ρgV* (2)

Here, *p* = Cu specimen perimeter, *g* = 9.81 m/s^2^, *V* = Cu specimen dipped volume in the solder bath, *ρ* = density of solder, and θ is the wetting angle. For maximum wettability, the wetting angle is zero. Consequently, Equation (2) simplifies to:

*F*_wd_ = *pσ_LV_* − *ρgV* (3)

Here, *F*_wd_ denotes the maximum withdrawal force, and the surface tension is computed using Equations (2) and (3):

*σ_LV_* = (*F*_wd_ + *ρgV*)/*p* (4)

Five measurements of wetting time (ZCT = zero cross time, time when the curve crosses the zero wetting force line) were recorded for each sample and averaged out.

### 2.3. Tensile Strength Test

**Figure 2:**
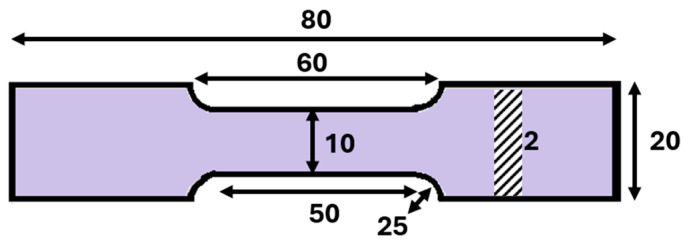
Scheme for the tensile tests. Units are in mm.

Tensile tests were conducted employing a universal testing machine (UTM, Norwood, MA, USA) with a strain rate of 3 × 10^−3^ s^−1^. The identical as-cast specimens were melted in a big pot at 300 °C and poured in a tensile mould of inside dimensions shown in Figure 2. As soon as the tensile mould is cooled down, it is opened to get the well-defined tensile samples. Three identical samples were produced for each solder composition in this work. The tensile specimens depicted in Figure 2 featured a dog-bone flat shape with a thickness of 2 mm and a gauge length of 20 mm, derived from the as-cast solder specimen.

Five parallel samples were subjected to testing for each composition. The ultimate tensile strength (UTS) and percentage elongation (%El) were determined from the stress strain diagrams.

### 2.4. Taguchi Optimization

With Minitab 19 software, the various soldering parameters targeted to two variables (wt.% Bi, wt.% Sb) and three levels (0, 1, 2 wt.%) were assessed by following the factorial DOE. The parameters were the wt.% Bi, wt.% Sb, melting point (°C), wetting time (ZCT), tensile strength (UTS), and El%. The notations of various samples used in the present work are listed in Table 1. The S/N ratio for the variables was optimized using “the smaller, the better” criterion.

## 3. Results and Discussion

In this Section, we discuss how the addition of Sb and Bi changes the microstructure, IMCs thickness which consequently affects the wettability, melting, and tensile characteristics of the SAC alloy. Detailed observations and explanations are provided to highlight the significance of these findings. We have also used Taguchi analysis to understand the effect of fraction of Bi and Sb (two-factors) and their levels (0, 1, and 2 wt.%) on these properties.

### 3.1. Microstructure

**Figure 3:**
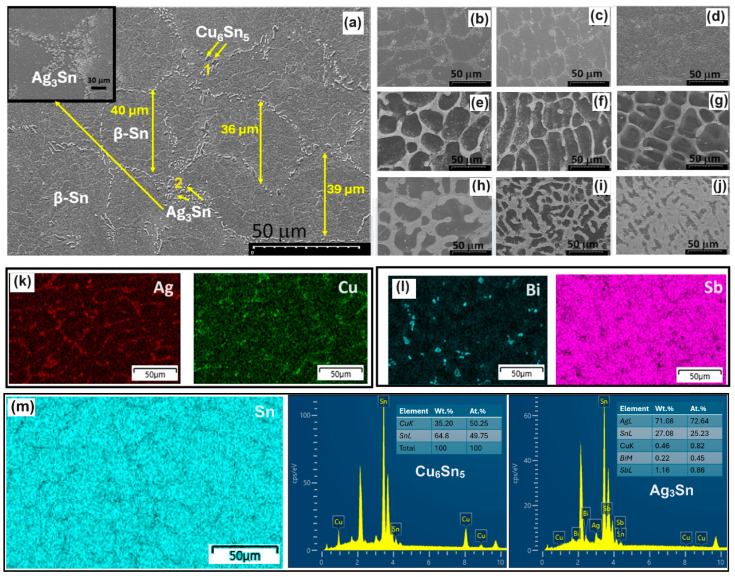
SEM images SAC105/(Bi, Sb) alloys. (**a**) SAC105, (**b**) SAC105-1Bi, (**c**) SAC105-2Bi, (**d**) SAC105-4Bi, (**e**) SAC105-1Sb, (**f**) SAC105-2Sb, (**g**) SAC105-4Sb, (**h**) SAC105-2Bi-2Sb, (**i**) SAC105-2Bi-4Sb, and (**j**) SAC105-4Bi-2Sb. The EDS mapping analysis of SAC105 and SAC105-4Bi-2Sb are given in (**k**,**l**). The quantitative EDS point analysis of Cu_6_Sn_5_ and Ag_3_Sn IMCs (point 1 and 2) is shown in (**m**) with matrix Sn map.

Figure 3 displays the morphology of SAC105 and SAC105/(Sb, Bi) alloys at various Sb and Bi contents. The SAC105 alloy exhibits a typical SAC morphology comprising eutectic β-Sn + Ag_3_Sn phases and primary β-Sn (Figure 3a). In the monolithic SAC105 specimen, the eutectic region, the size of β-Sn grains and IMCs (Ag_3_Sn, Cu_6_Sn_5_) is relatively large (Figure 3a). The mean β-Sn grain size is around 38 µm while the IMCs have a size of 3.0 ± 0.5 µm. With the addition of additive elements (Bi, Sb) to SAC105, the microstructure undergoes refinement, evidenced by a noticeable reduction in β-Sn grain area and IMC thickness (Figure 3b–j). The grain size decreases from 38 µm to 28 µm for SAC105-1Bi, 24 µm for SAC105-3Bi, and 26 µm for SAC105-5Bi. It is seen that the addition of Bi in SAC105 brings a 36.8% decrease in grain area for SAC105-3Bi. Similarly, the grain size decreases from 38 µm to 31 µm for SAC105-1Sb, 29 µm for SAC105-3Sb, and 33 µm for SAC105-5Sb. In this case of Sb, there is slight change (23.6% decrease) observed for SAC105-3Sb. However, when Bi and Sb are added simultaneously in SAC105, the grain area decreases to 18 µm for SAC105-1Bi-1Sb, 11 µm for SAC105-2Bi-4Sb, and 9 µm for SAC105-4Bi-2Sb. Thus, a combined addition of Bi and Sb brings maximum change in grain refinement up to 76% for SAC105-4Bi-2Sb. The addition of Sb and Bi (>4%) further reduced grain size but increased IMCs in the microstructure. The EDS maps confirm the presence of Ag_3_Sn and Cu_6_Sn_5_ in SAC105 (Figure 3k). In addition, the Bi is found to be segregated around the grain boundaries while Sb is dissolved into the β-Sn grains to form the solid solution (Figure 3l). The point EDS analysis showed the presence of Cu_6_Sn_5_ and Ag_3_Sn IMCs in the matrix (Figure 3m). In our several experiments involving a Bi-containing SAC alloy, it was revealed that the Bi concentration deviated from the local equilibrium as expected from the phase diagram, which indicated a higher local concentrations and lesser liquid concentration than expected (Figure 3l). The reason for this precipitation may be ascribed to the fact that segregation factor of Bi in Sn low angle grain boundaries is high enough to be discharged into adjacent grains for a Sn-Bi system [28].

**Figure 4:**
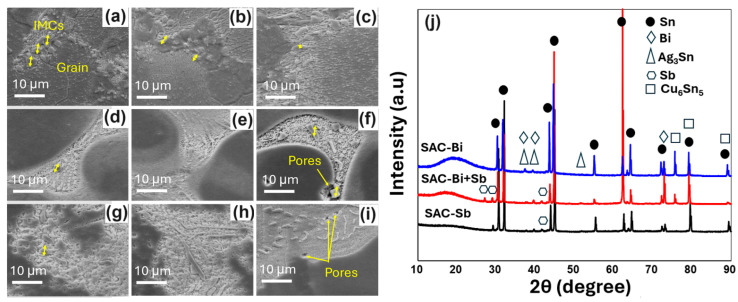
High resolution SEM images SAC105/(Bi, Sb) alloys. (**a**) SAC105-1Bi, (**b**) SAC105-2Bi, (**c**) SAC105-4Bi, (**d**) SAC105-1Sb, (**e**) SAC105-2Sb, (**f**) SAC105-4Sb, (**g**) SAC105-1Bi-1Sb, (**h**) SAC105-2Bi-2Sb, and (**i**) SAC105-4Bi-4Sb, (**j**) XRD pattern of SAC105, SAC105-Bi, and SAC105-Bi-Sb alloy.

The compositional EDS analysis of the various samples SAC105 and SAC105/(Bi, Sb) shows that Bi and Sb disperse more evenly in the Sn matrix while restricting the growth of IMCs (Figure 4a–i). The major IMCs in these samples were found to be Ag_3_Sn compounds, as confirmed from the XRD measurements (Figure 4j). To compare the effects of Bi and Sb in SAC alloy, we observed the microstructure at higher resolution as shown in Figure 4. As the individual component increased, the thickness of the IMC shows slight reduction at 1wt%Bi or Sb but increases at higher fractions of Bi or Sb (Figure 4a–f). However, when Bi and Sb are added simultaneously in SAC105, the IMC layer was substantially thinned down in SAC105-2Bi-2Sb sample as compared to the rest (Figure 4g–i).

The quantitative measurements of the IMC thicknesses are summarized in Table 2. It is shown that the extent of IMC increments is more in Bi-modified SAC105 (3 to 3.6 µm) as compared to Sb-modified SAC105 (3.0 to 2.9 µm).

In comparison, the combined effect of Bi + Sb showed a maximum refinement in IMC thickness, and it stays close to 3 µm (3.0 to 2.7 µm). Gradual substitution of Bi for Sb refines the microstructure, and the IMCs (Ag_3_Sn and Cu_6_Sn_5_) are more uniformly distributed in the SAC105 matrix. Simultaneously, due to the substantial size mismatch between Bi, Sb, and the Sn matrix (Table 3), the solution hardening effect resulting from the addition of dual Bi and Sn becomes more pronounced.

As shown in Table 3, from the atomic size of the added elements in SAC105 alloy, it is evident that the atomic size mismatch between Bi and Sn surpasses that of the Sb and Sn. Furthermore, the effective atomic mismatch between (Bi, Sb) and Sn is the maximum that plays a role in the superior strengthening effect. The microstructural investigations show that incorporation of Bi and Sb > 2 wt.% increases the IMC thickness and grain size of the alloy [29,30]. Therefore, based on these observations, we fixed the two factors (Bi, Sb) up to the three levels (0, 1, 2 wt.%) for statistical analysis and designed a 2 × 3 factorial DOE.

### 3.2. Wettability

**Figure 5:**
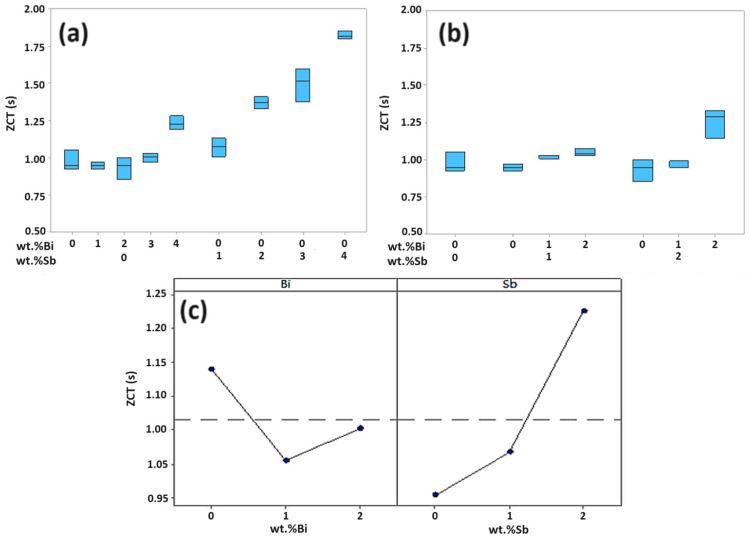
Effect of Bi, Sb on wetting time in SAC105 alloy. (**a**) Effect of different Bi and Sb on ZCT for SAC105 alloy. (**b**) Effect of Bi + Sb on ZCT in SAC105. (**c**) Interaction plot for Bi and Sb on ZCT in SAC105 solder alloy.

The wetting balance test results are shown in terms of zero cross time (ZCT) (Figure 5). A smaller wetting force signifies an increased ZCT (poor wetting) [12]. It is observed that the ZCT is hardly changed (<1.00 s) until 2 wt.% Bi is added to the SAC105 (Figure 5a). The addition of Bi > 0.2 wt.% in SAC105 increases the ZCT to 1.25 s, which indicates the sample takes enough time to wet. In other words, the wetting is degraded severely at higher fractions of Bi (4 wt.%) in SAC105 (Figure 5a). A similar behaviour is seen when Sb is added to the SAC105 samples; however, Sb-containing samples have higher ZCT (1.1 to 1.81 s) as compared to Bi-containing samples (<1.25 s). The addition of an optimum amount of Bi and Sb in the SAC105 matrix reduces the interface surface energy between the solder and Cu coupon, thereby enhancing wetting. To elaborate, the interfacial energy refers to the energy of the contact boundary between two phases, such as a solid and a liquid. When Sb and Bi are added to the brazing metal, they alter the physico-chemical properties of the interfacial boundary and reduce the interface energy. A lower interface energy typically leads to better wettability of the liquid solder on the solid surface. This enhancement in wettability ensures a more uniform and stronger bond between the materials being joined. The reduced interface energy allows the liquid solder to flow more easily and adhere more effectively to the surfaces, resulting in higher-quality brazed joints. It is noteworthy that the ZCT deteriorates in the case of Bi and Sb (both > 2 wt.%) in SAC105 solder. This behaviour is attributed to the higher amount of Bi and Sb, causing them to segregate in melt. This result aligns with findings from other reports on Pb-free solders in the past [14,17,19].

Monolithic SAC105 exhibits a much higher ZCT compared to SAC105/(Bi, Sn) alloys. For instance, ZCT for monolithic SAC105 is 0.981 s. It stays around 1.01 s for SAC105-1Bi-1Sb alloys. The ZCT is maximum for SAC1052Bi-2Sb but it is still close to 1.25 s (Figure 5b). Optimal wetting behaviour is observed for dual alloying additions (Bi, Sb: 2, 2) in the SAC105 solder matrix. This is also clear from the interaction plot of Bi- and Sb-containing SAC105 on ZCT. The de-wetting process is generally associated with the segregation of Bi and Sb at higher fractions in the matrix due to the reduced melt fluidity. This behaviour can impede the flow of liquid solder aligning with observations from previous reports [14,19]. According to Liu et al., it is reported that the addition of Bi reduces the melting point of the alloy and increases spreadability because it is an interfacial active element that promotes diffusion by reducing the interfacial tension of the liquid solder [31]. According to Pavel et al., as the Sb fraction rises above 2%, IMCs produced due to excessive reaction between solder and Cu become more common in soldering. This has been reported to be effective in preventing spread [32,33]. Therefore, the optimal Sb content for wetting rate appears to be approximately 2%.

The results were subjected to ANOVA using Minitab to understand the wetting behaviour. The wetting test (ZCT data) when Bi and Sb components were added independently into the SAC105 are shown in Figure 5c. When Bi content is 2 wt.%, the wetting time decreased slightly but tended to increase at higher contents, while as the Sb content increased, the wetting time clearly increased. The appropriate addition of Bi can slightly reduce the wetting time if Bi is less than 2 wt.%. However, if the Bi content increases excessively, a thick Bi-rich IMC layer is formed, which reduces the adhesion of the solder to the Cu coupon and increases the wetting time. Therefore, it is believed that the wetting time initially decreases as the Bi content increases, but increases after a certain amount due to the formation of a thick Bi-rich layer [14,19].

When the Bi-Sb alloy element is added at 1 wt.%, the wetting time increases gently as the Sb content increases, and the increase in wetting time is not as large as when Sb is added as a single component. This is thought to be due to the influence of Bi. When Bi is 2 wt.% and Sb is 2 wt.%, the wetting time increases significantly and shows similar characteristics to 2 wt.% Sb as a single component. Table 4 is the result of ANOVA on three levels (0, 1, 2 wt.%) of the ZCT experiment. Both Bi and Sb have *p*-values below 0.05 at wetting time. It is a significant factor, and the *p*-value of Bi + Sb is 0.000, which is less than 0.05, indicating that there is an interaction between Bi and Sb. The model summary shows that the one-dimensional regression model has an explanatory power of 91.50%. The optimal combination of Bi and Sb for wetting time characteristics is when the Bi content is 2 wt.% and the Sb content is 2 wt.% with a degree of optimization is 80.88%.

### 3.3. Melting Point

**Figure 6:**
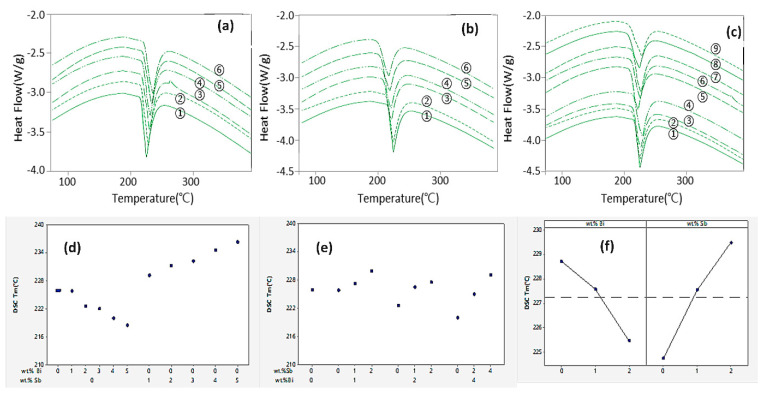
DSC curves of (**a**) different SAC105/Bi alloys (Bi: 0, 1, 2, 3, 4, 5 wt.% corresponding to samples (1) to (6), respectively), (**b**) SAC105/Sb (Sb: 0, 1, 2, 3, 4, 5 wt.% corresponding to samples (1) to (6), respectively), and (**c**) SAC105/Sb + Bi (Bi: 0, 1, 2, 3, 4 wt.%; Sb: 1, 2, 3, 4 wt.% corresponding to samples (1) to (9), respectively). (**d**) Effect of different Bi and Sb contents in SAC105, (**e**) effect of Bi + Sb contents in SAC105, and (**f**) interaction plot for Bi and Sb on melting point of SAC105 solder alloy.

Figure 6a–c illustrates the DSC curves of SAC105 and SAC105/(Bi, Sb) (*x* = 0, 1, 2, 3, 4 wt.%) solders. The melting point changes when the Bi and Sb components are added to the SAC105 alloy. As the Bi content rises from 1 to 5 wt.%, the melting point tends to decrease while the melting point tends to increase as the Sb content increases from 1 to 5 wt.%. The melting point of SAC105 increases when Sb is added to 1 to 4 wt.% Bi.

Table 5 is the result of ANOVA for melting points at three levels (0, 1, 2 wt.%). Both Bi and Sb are significant factors affecting the melting point with a *p*-value of 0.05 or less, and there is no interaction of Bi + Sb. The model summary shows that the 1D regression model has an explanatory power of 96.51%.

The melting point is appreciably depressed to 222.55 °C when the Bi content is 2 wt.%, which is the maximum of the experimental range, and the Sb content is 0 wt.%. The influence of Sb can be seen to be greater. In the SAC105-Sb and SAC105-Bi-Sb alloys, the increase in Sb only increased the liquidus temperature. During solidification, Bi and Sb particles offer multiple nucleation sites, reduce undercooling, and favour the melting point depression. This suggests that the effect of Bi on reducing melting point is more significant than Sb, thus improving the solder alloy melting point. In contrast, Bi is inert to the other elements, dispersing in the alloy matrix and contributing to structural uniformity [34,35,36]. Although alloying elements can inevitably increase the pasty range, the melting range of SAC105-2Sb-2Bi expanded less compared to other solder alloys, indicating relatively better melting characteristics.

### 3.4. Tensile Test

**Figure 7:**
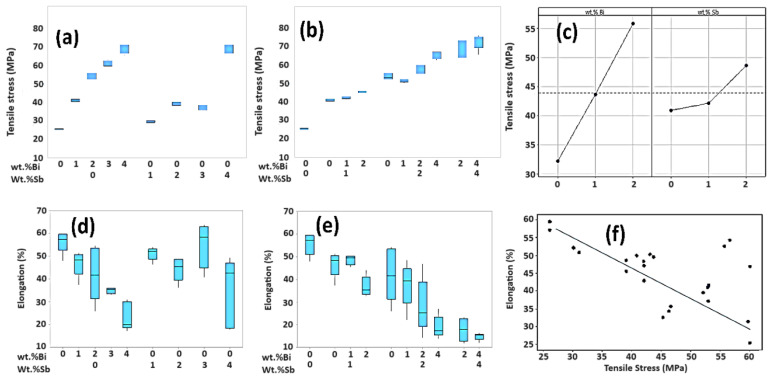
Mechanical properties. Tensile strength of (**a**) different SAC105, SAC105/(Bi, Sb) alloys and (**b**) different Bi + Sb contents in SAC105. (**c**) Interaction plot for Bi and Sb on UTS of SAC105 solder alloy. El% of (**d**) different SAC105, SAC105/(Bi, Sb) alloys and (**e**) different Bi + Sb contents in SAC105. (**f**) Correlation of UTS and El%.

The mechanical properties of SAC-105-Bi, SAC105-Sb, and SAC105-(Bi + Sb) alloys such as UTS and %El are presented in Figure 7a,b. It is seen that SAC105 exhibits the lowest UTS (25.2 MPa) but displays excellent %El (58%). The introduction of 2 wt.% Sb to SAC105 enhances the UTS, as Sb forms solid solutions in Sn, refining the Ag_3_Sn IMCs layer. This results in increased strength, albeit with a slight decrease in %El [22,37].

The addition of up to 4 wt.%Bi and 4 wt.%Sb to SAC105 significantly improves the UTS, nearly doubling it compared to SAC105, SAC/Bi, and SAC/Sb alone. However, due to the adverse effects of coarse IMCs on soldering, %El is substantially reduced. In comparison, the dual additives (4Bi-4Sb (wt.%)) effectively reduce the size of IMCs, distributing them in the matrix, which leads to a superior UTS (>70 MPa) while the %El is decreased to minimum. The UTS increases with Bi but decreases a bit with Sb (Figure 7c). These improved UTS are attributed to the synergy of Bi and Sb where Sb forms a solid solution with Sn while Bi acts as a dispersing phase in the SAC105 solder alloy, reducing Ag_3_Sn IMCs [22,23].

In the samples containing Sb and Bi together, the tensile stress tends to increase more significantly compared to when Bi is added alone. The tensile stress distribution was analysed for samples with 1–4 wt.%Bi, 1–4 wt.%Sb, and combinations of 1–1 wt.% to 2–2 wt.%Bi + Sb. For the Bi + Sb mixtures, the effects of Bi and Sb counteract each other, resulting in intermediate tensile strength characteristics.

Figure 7d,e shows the elongation characteristics when 0 to 4 wt.% of Bi and Sb components are added, respectively. The elongation tends to decrease as the Bi and Sb contents increase. Figure 7f shows the elongation characteristics when 0 to 4 wt.% of Bi + Sb is added. As the Bi + Sb mixing component increases, the %El tends to decrease. The condition for balanced UTS and %El is when the Bi and Sb both are 2 wt.%; the UTS is 58 MPa at 25% El%. The mechanical properties at 4 wt.%Bi and Sb are not desirable due to excessive strength but least %El value. Table 6 is the result of ANOVA for %El at three levels (0, 1, 2 wt.%). Both Bi and Sb have a *p*-value of 0.05 or less, showing there is no interaction of Bi + Sb. The conditions for maximizing UTS and %El are when the Bi content is 2 wt.%, the maximum of the experimental range, and the Sb content is 0 wt.%, the minimum UTS is 52.87 MPa, and the %El 47.53% (Figure 7f). In this analysis result, when 2 wt.%Bi and 2 wt.% Sb are combined, the melting point according to DSC analysis is 222.55 °C, wetting time is 0.94 s, %El is 49.51%, UTS is 55.09 MPa, and there is a satisfaction index of 0.71 (=71%). Therefore, it can be concluded that the combination of Bi + Sb in SAC105-Bi-Sb solder alloy through multi-optimization analysis of experimental results should not exceed 2 wt.%.

## 4. Conclusions

1. The incorporation of Bi and Sb slightly depressed the melting point of the SAC105 alloy. However, when the Bi and Sb are added in combination into SAC105, the melting point showed a drastic reduction which is beneficial to low temperature soldering.
2. The wetting balance tests demonstrated that the addition of 2 wt.% of Bi and Sb enhanced the wetting of SAC105 alloys. The combined addition of Bi + Sb showed a more pronounced wetting up to 2 wt.%Sb and Bi.
3. The addition of 2 wt.% of Bi and Sb into SAC105 slowed the growth of Sn grains and Ag_3_Sn IMCs without forming any Bi- or Sb-compounds. The β-Sn grain size and IMCs are refined with the addition of Bi or Sb, Bi being a stronger grain refiner than Sb.
4. This study demonstrates that the mechanical properties, UTS and %El, could be significantly controlled when Bi and Sb are embedded into the SAC105 alloy. However, it should be noted that the fraction of Bi and Sb should not exceed 2 wt.% for better realization of properties.
5. The Taguchi optimization method predicted a set of combination of properties for better realization of soldering properties to be 2 wt.%Bi and 0 wt.%Sb; ZCT = 0.9 s, wetting point of 222.55 °C, UTS of 55 MPa, and %El of 50%.

## Figures and Tables

**Table 1:** Experimental plan for Bi and Sb addition in SAC105 alloy.

S. No.	Sample	wt.%Bi	S. No.	Sample	wt.%Sb	S. No.	Sample	wt.%Bi, wt.%Sb
1	SAC105	0				12	SAC105-1Bi-1Sb	1, 1
2	SAC105-1Bi	1	7	SAC105-1Sb	1	13	SAC105-1Bi-2Sb	1, 2
3	SAC105-2Bi	2	8	SAC105-2Sb	2	14	SAC105-2Bi-1Sb	2, 1
4	SAC105-3Bi	3	9	SAC105-3Sb	3	15	SAC105-2Bi-2Sb	2, 2
5	SAC105-4Bi	4	10	SAC105-4Sb	4	16	SAC105-2Bi-4Sb	2, 4
6	SAC105-5Bi	5	11	SAC105-5Sb	5	17	SAC105-4Bi-2Sb	4, 2
						18	SAC105-4Bi-4Sb	4, 4

**Table 2:** Thickness of IMCs in SAC105 according to the different Bi and Sb fractions.

Samples	IMCs Thickness
SAC105	2.9 ± 0.45 µm
SAC105-1Bi	3.0± 0.51 µm
SAC105-2Bi	3.4 ± 0.57 µm
SAC105-4Bi	3.6 ± 0.69 µm
SAC105-1Sb	2.8 ± 0.35 µm
SAC105-2Sb	2.7 ± 0.29 µm
SAC105-4Sb	2.9 ± 0.32 µm
SAC105-2Bi-2Sb	2.9 ± 0.35 µm
SAC105-2Bi-4Sb	2.6 ± 0.26 µm
SAC105-4Bi-2Sb	2.7 ± 0.27 µm

**Table 3:** The atomic size of various elements in this work.

Elements	Sn	Ag	Cu	Bi	Sb
**Atomic radius (nm)**	0.158	0.144	0.128	0.170	0.160

**Table 4:** ANOVA table of wetting time for 2 factors (Bi, Sb) and 3 levels (0, 1, 2 wt.%).

Sources	DF	Adj SS	Adj MS	F-Value	*p*-Value
**Model**	8	0.54	0.06	24.23	0.000
Linear	4	0.44	0.11	39.97	0.000
Bi	2	0.08	0.04	15.04	0.000
Sb	2	0.36	0.18	64.91	0.000
Bi + Sb	4	0.09	0.02	8.49	0.000
**Model Summary**					
S	R-square	R-S (modify)	R-S (forecast)		
0.05	91.50%	87.73%	80.88%		

**Table 5:** ANOVA of melting point for 2 factors (Bi, Sb) and 3 levels (0, 1, 2 wt.%).

Sources	DF	Adj SS	Adj MS	F-Value	*p*-Value
**Model**	4	50.342	12.5854	27.66	0.004
Linear	4	50.342	12.5854	27.66	0.004
Bi	2	16.22	8.11	15.04	0.010
Sb	2	34.121	17.0607	64.91	0.003
Bi + Sb	4	1.38	2.45	12.56	0.002
**Model Summary**					
S	R-square	R-S (modify)	R-S (forecast)		
0.67452	96.51%	93.02%	82.34%		

**Table 6:** ANOVA of tensile strength and elongation for 2 factors (Bi, Sb) and 3 levels (0, 1, 2 wt.%).

Tensile Strength					
Sources	DF	Adj SS	Adj MS	F-value	*p*-value
Model	4	2852.71	713.18	125.64	0.000
Linear	4	2852.71	713.18	125.64	0.000
Bi	2	2540.8	1270.4	223.8	0.000
Sb	2	311.91	155.96	27.47	0.000
Bi + Sb	4	124.88	5.68	30.08	0.000
**Model Summary—Tensile strength**					
S	R-square	R-S (modify)	R-S (forecast)		
2.38254	95.81%	95.04%	93.68%		
**Elongation**					
**Sources**	**DF**	**Adj SS**	**Adj MS**	**F-value**	***p*** **-value**
Model	4	1492.8	373.2	15.6	0.000
Linear	4	1492.8	373.2	15.6	0.000
Bi	2	575.3	287.66	12.03	0.000
Sb	2	917.5	458.73	19.18	0.000
Bi + Sb	4	526.2	23.92	9.84	0.000
**Model Summary—Elongation**					
S	R-square	R-S (modify)	R-S (forecast)		
1.35492	96.24%	96.09%	95.74%		

## Data Availability

Data are contained within the article.
